# Quantifying the Persistence of Vaccine-Related T Cell Epitopes in Circulating Swine Influenza A Strains from 2013–2017

**DOI:** 10.3390/vaccines9050468

**Published:** 2021-05-06

**Authors:** Swan Tan, Andres Hazaet Gutiérrez, Phillip Charles Gauger, Tanja Opriessnig, Justin Bahl, Leonard Moise, Anne Searls De Groot

**Affiliations:** 1 Department of Infectious Diseases, University of Georgia, Athens, GA 30602, USA; swan.tan@uga.edu (S.T.); JUSTIN.BAHL@uga.edu (J.B.); 2 Center for Vaccines and Immunology, University of Georgia, Athens, GA 30602, USA; lmoise@uga.edu or; 3 EpiVax Inc., Providence, RI 02909, USA; agutierrez@epivax.com; 4 Department of Veterinary Diagnostic and Production Animal Medicine, College of Veterinary Medicine, Iowa State University, Ames, IA 50011, USA; pcgauger@iastate.edu (P.C.G.); or Tanja.Opriessnig@roslin.ed.ac.uk (T.O.); 5 Roslin Institute, University of Edinburgh, Midlothian EH25 9RG, UK

**Keywords:** swine IAV, immunoinformatics, T cell epitope conservation

## Abstract

When swine flu vaccines and circulating influenza A virus (IAV) strains are poorly matched, vaccine-induced antibodies may not protect from infection. Highly conserved T cell epitopes may, however, have a disease-mitigating effect. The degree of T cell epitope conservation among circulating strains and vaccine strains can vary, which may also explain differences in vaccine efficacy. Here, we evaluate a previously developed conserved T cell epitope-based vaccine and determine the persistence of T cell epitope conservation over time. We used a pair-wise homology score to define the conservation between the vaccine’s swine leukocyte antigen (SLA) class I and II-restricted epitopes and T cell epitopes found in 1272 swine IAV strains sequenced between 2013 and 2017. Twenty-four of the 48 total T cell epitopes included in the epitope-based vaccine were highly conserved and found in >1000 circulating swine IAV strains over the 5-year period. In contrast, commercial swine IAV vaccines developed in 2013 exhibited a declining conservation with the circulating IAV strains over the same 5-year period. Conserved T cell epitope vaccines may be a useful adjunct for commercial swine flu vaccines and to improve protection against influenza when antibodies are not cross-reactive.

## 1. Introduction

When a new strain of pathogen emerges, the first question asked is often whether existing vaccines might be effective against it. In the past, experts have relied on examining the humoral immune response by using antibody assays to determine the potential of existing vaccines to cross-protect [1]. It is now well established that cell-mediated immunity (CMI) contributes to the protection against severe disease even in the absence of antibody response [2,3,4,5]. CMI involves cytotoxic T lymphocytes (CTL) and T helper (Th) lymphocytes, which are triggered to respond when their T cell receptors (TCR) recognize T cell epitopes presented by class I or class II major histocompatibility complex (MHC) molecules on the surface of antigen presenting cells or infected cells [6].

In humans, immune responses to conserved T cell epitopes may result in reduced morbidity, despite the lack of cross-reactive antibody to the new strain [7,8,9]. This is supported by a case-controlled study that investigated the association of the pandemic IAV H1N1 2009 infections with 2008–2009 seasonal trivalent inactivated flu vaccination [10]. Previous seasonal vaccination protected against pandemic H1N1, despite the lack of antibody protection. Using the immunoinformatic tools available to us at the time, we defined T cell epitopes that were present in the newly emergent strain (pH1N1 A/California/04/2009; GenBank accession numbers ACP41105 for hemagglutinin or HA and ACP41107 for the neuraminidase or NA), and highly conserved in the existing seasonal influenza vaccine (containing H1N1 A/Brisbane/59/2007; GenBank accession numbers ACA28844 for HA and ACA28847 for NA) [11]. The in silico analysis demonstrated that despite the lack of antibody cross-reactivity, more than 50% of T cell epitopes in the novel pH1N1 virus were also present in the seasonal vaccine, supporting the concept that pre-existing T cell response due to vaccination or exposure may have protected in the absence of protective antibody response.

Swine experimental studies have also shown that pigs were protected from heterologous infection and challenge between ‘avian-like’ H1N1 and 2009 pandemic H1N1 lineages in the absence of cross-reactive antibodies, establishing the role of cross-reacting T cells [12,13]. In concurrence, researchers were able to identify cross-reacting CD8 T cell epitopes in pigs and nucleoprotein (NP)-specific CD8 T cells were induced following immunization by aerosol [14,15]. Prospective animal studies also confirmed that seasonal H1N1 vaccines that did not induce cross-reactive antibody responses, but induced cross-reactive T cell responses, did not protect against the pandemic pH1N1 infection, but greatly reduced morbidity, mortality, virus replication, and viral shedding [16]. Thus, T cell epitopes can be conserved between both human and swine vaccines and emerging influenza strains and have been shown to contribute to protection.

Antigenic shift and drift are significant challenges not only to human seasonal vaccination but also to effective swine flu vaccination over time. The segmented IAV genome allows for the antigenic shift by reassortment of RNA segments from different viral strains, generating novel viruses [17]. The antigenic drift that is due to the gradual accumulation of mutations in the HA and NA surface antigens over time also contributes to the remarkable diversity of IAVs co-circulating among swine populations. This sequence-level diversity can impact the T cell response since even single amino acid modifications to T cell epitopes can reduce human leukocyte antigen (HLA) binding or T cell recognition, leading to viral escape and viral camouflage [18,19] that contribute to a lower vaccine efficacy. An additional problem facing swine influenza vaccine developers is the high diversity of circulating IAV genotypes impacting individual pork farms each year [17], making it difficult to know whether a given commercial vaccine will be protective.

To address these challenges in pigs, we applied a previously developed computational method for estimating the degree of epitope conservation between vaccines and outbreak strains. Rather than focus on sequence identity, this algorithm identifies individual epitopes, and searches for epitope pairs that share MHC-binding properties and have identical TCR-facing residues, while allowing for amino acid variability at the T cell agretope (the HLA-binding-pocket facing amino acid residues). For swine IAV, the first step is to use SLA prediction matrices (PigMatrix) [20], and once an SLA-binding T cell epitope is predicted, JanusMatrix is applied to isolate the TCR-facing residues for comparison with similar SLA-binding epitopes in other circulating influenza strains [21,22]. The third step is to use the Epitope Content Comparison (EpiCC) tool, which compiles the similarities and differences between the T cell epitopes in the vaccine and the circulating strain, assigning a score that reflects the degree of conservation [23].

Here, we compare a computationally designed swine flu vaccine based on conserved T cell epitopes called multi-epitope vaccine (MEpiV) with commercially available inactivated full strain vaccines using the computational algorithms described above. The MEpiV is composed of immunoinformatic-identified conserved SLA class I and class II epitopes assembled head-to-tail as class I and class II poly-epitope genes and formulated for delivery in a DNA vaccine vector [24]. The MEpiV was previously shown to be protective in a heterologous prime-boost vaccination and challenge study when combined with the whole-inactivated vaccine [25]. In this study, we determine if the conserved T cell epitope-based vaccine would maintain conservation with circulating strain T cell epitopes over time.

To evaluate the T cell epitope conservation for the MEpiV vaccine and to compare the conservation of the epitopes selected in 2013 to circulating strains for subsequent years, we used the HA sequence of seasonal inactivated swine flu vaccines as a benchmark for comparison. Then, we applied EpiCC and demonstrated that the MEpiV vaccine designed using computational tools in 2013 maintains >50% conservation with circulating strains over a 5-year period. As can be expected, EpiCC also indicated that T cell epitopes in commercial seasonal vaccines are less well conserved over the same time period. This evaluation of vaccines using EpiCC shows the approach to understanding T cell epitope conservation and the utility of the tool for comparing vaccines against emerging influenza strains. The analysis also reinforces the utility of designing influenza vaccines based on highly conserved epitopes from circulating viral strains, as these epitopes may be conserved over time.

## 2. Materials and Methods

### 2.1. Datasets

The sequences of all available H1N1, H1N2, and H3N2 swine IAV genomes circulating in the United States during the 5-year period 2013–2017 were obtained from the NIAID Influenza Research Database (https://www.fludb.org/, accessed on 29 April 2021) [26]. All of the genome sequences were downloaded and pre-processed to remove partial and duplicated sequences. The final set of 1272 whole genome sequences were translated into protein sequences and were compared to the epitope-based DNA vaccine, MEpiV (Table S1) using an immunoinformatic approach as described below. In order to further evaluate the conservation of MEpiV, the sequence from two standard inactivated swine IAV vaccine antigens (FluSureXP 2016) were included for comparison. HA sequences of inactivated swine IAV vaccine comprised of one H1N1, one H1N2, and two H3N2 strains were provided by Zoetis to facilitate the comparison of T cell epitope conservation in the epitope-based and inactivated virus vaccine, applying the same immunoinformatic analysis pipeline. The level of conservation for each of the vaccines (HA from H1N1 to H3N2, the MEpiV as compared to a representative set of IAV strains for each year was measured relative to 2013 to obtain relative changes in the number of T cell epitopes that were conserved.

### 2.2. Immunoinformatic Tools

**Figure 1:**
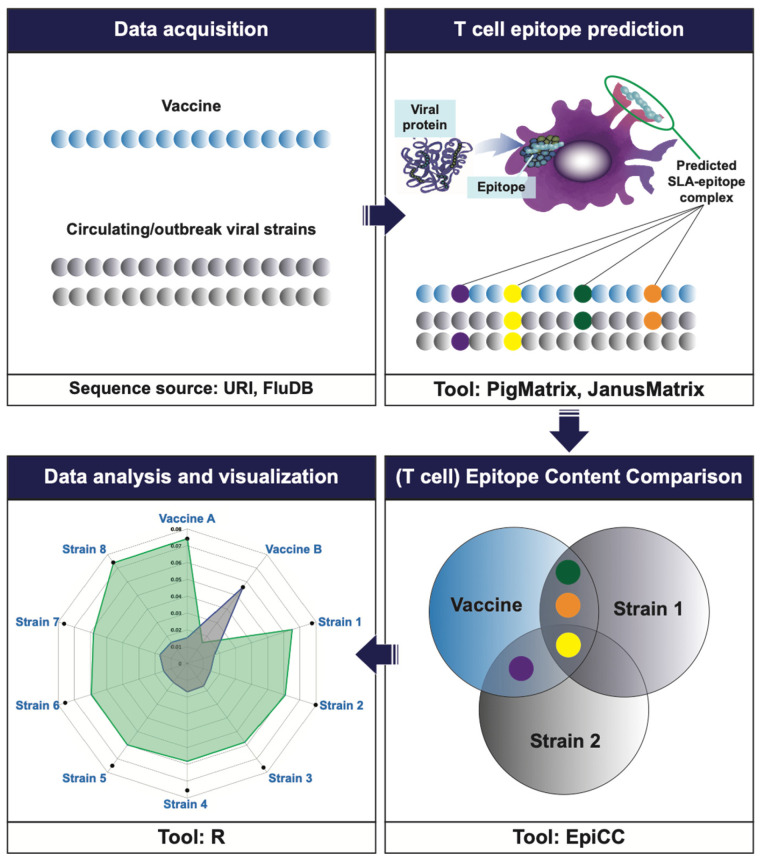
Workflow for the typical EpiCC analysis. The vaccine sequence of interest (here, MEpiV) and circulating pathogen strains (swine IAV, in this example) are retrieved and pre-processed prior to performing the EpiCC analysis. T cell epitopes are identified in the vaccine and circulating strains (colored beads) using EpiMatrix (for HLA restricted human T cell epitopes) or PigMatrix (for SLA-restricted T cell epitopes). Once all the epitopes are identified, a comparison and quantification of the T cell epitopes is performed using EpiCC. An overall EpiCC score (area under the curve) is calculated for the combined class I and II epitopes for each vaccine/strain comparison. Greater AUC scores indicate higher numbers of conserved T cell epitopes. EpiCC scores can be compared and contrasted for the selected vaccines (here, MEpiV versus seasonal whole inactivated swine IAV vaccines).

Three separate algorithms were used to evaluate the conservation of vaccine epitopes contained in the target vaccine against the complete set of swine IAV sequences: (1) PigMatrix, which defines T cell epitopes for swine class I and class II epitopes, (2) JanusMatrix (JMX), a tool for identifying epitopes that can be compared between strains by looking for epitopes that bind to the same allele and have conserved TCR facing residues which can be used to compare strains, and (3) EpiCC, the T cell epitope content comparison algorithm utilizes results generated from PigMatrix and JMX and produces an overall score for class I and/or class II epitopes on a whole antigen level to enable pairwise comparisons between circulating IAV and vaccine strains (See Figure 1). A total of 1272 pairwise comparisons were performed, comparing each 9-mer sequence for a possible conservation of SLA binding and TCR face, between MEpiV vaccine and circulating strains. EpiCC examines all of the epitopes in a vaccine against all of the epitopes in a given strain and produces an overall score for all class I or class II epitopes for each strain sequence. In addition to EpiCC, we used JanusMatrix to perform the same comparison on an epitope-by-epitope basis for the 28 class I and 20 class II epitopes in the vaccine.

### 2.3. T Cell Epitope Prediction Using PigMatrix

Using the pocket profile method and well-defined EpiMatrix binding preferences for human MHC pockets, we developed PigMatrix prediction matrices as previously described [20,21]. Matrices were designed based on the binding preferences of the best-matched human leukocyte antigen (HLA) pocket for each SLA pocket. The contact residues involved in the binding pockets were defined from crystal structures of SLA or HLA supertype alleles for class I and II, respectively. The allele selection was based on prior data indicating their prevalence in outbred swine populations [27,28], and frequencies determined using low-resolution haplotyping in a small number of pigs [24]. For low-resolution SLA-typing results where haplotype associations were not possible, XX01 alleles were selected. Matrices were constructed for SLA alleles with HLA binding pocket similarities above 85% to predict T cell epitope binding to class I (SLA-1*01:01, 1*04:01, 1*08:01, 1*12:01, 1*13:01, 2*01:01, 2*04:01, 2*05:01, 2*12:01, 3*04:01, 3*05:01, 3*06:01, 3*07:01) and class II (SLA-DRB1*01:01, 02:01, 04:01, 04:02, 06:01, 06:02, 07:01, and 10:01) SLA alleles. PigMatrix raw scores were standardized to Z-scores to compare potential epitopes across multiple SLA alleles. Peptides with Z-scores ≥1.64 (the top 5% of any given sample of 9-mers) were identified as likely to be SLA ligands.

### 2.4. Identification of Conserved Vaccine Epitopes in Different Circulating Swine IAV Subtypes

JanusMatrix (JMX) is another immunoinformatic algorithm, which was incorporated to prospectively identify conserved vaccine epitopes among prevalent swine IAV [22]. JMX is used to facilitate the epitope to an epitope-based comparison between swine IAV protein sequences and the vaccine strain. Conserved peptides at the TCR-face were searched against all the circulating strains and hence the presence of these peptides can be identified when there are matches in each individual strain.

### 2.5. T Cell Epitope Content Comparison (EpiCC) Analysis

In order to determine the conservation of the T cell epitopes in the MEpiV among the three-circulating swine IAV subtypes, we applied EpiCC to facilitate the pairwise comparison of protein sequences [23]. This method of comparison is based on an immunological property expressed in terms of T cell epitope content which incorporated JMX computation, rather than sequence identity. Shared (conserved) T cell epitopes between the vaccine target and the circulating swine IAV strains were evaluated. The assumption was based on the fact that given epitopes *i* and *j* from different strains (the circulating strain, s and the vaccine strain, v), cross-reactive memory T cells can be activated by epitopes with identical TCR-facing residues (TCR*f*) that bind to the same alleles. The potential cross-reactive of class I epitope is calculated by considering identical residues at positions 4, 5, 6, 7, and 8 and for class II, the calculation is taken into account by identical residues at positions 2, 3, 5, 7, and 8. Therefore, the probability to induce the cross T-cell immunity is computed based on the following equation and p stands for the probability for epitope binding to the HLA allele:

(1) $$S{\left(i,j\right)}_{a}=p{\left(i\right)}_{a}\cdot \;p{\left(j\right)}_{a}$$

By applying the above equation, the probability of cross T cell epitope between two strains s and v, can be calculated as follows:

(2) $$E\left(s,v\right)=\sum^{\;}i\in s,j\in v\sum^{\;}a\in A\cdot S{\left(i,j\right)}_{a}$$

To further compute the shared T cell epitope content between (conserved) two strains, i.e., EpiCC score, the following equation is applied:

(3) $$P\left(shared\right)=\frac{2\;\cdot E\left(s,v\right)}{E\left(s,s\right)+E\left(v,v\right)}$$

### 2.6. Area under the Curve (AUC) Computation

Given the fact that the complexity of multiple comparisons was done according to years and subtypes, the AUC calculation is applied to represent the T cell epitope conservation of a subtype in a year. EpiCC scores that were calculated for MEpiV and swine IAV sequences were plotted in a radar form (a line plot that is on circular orientation). The area under the radar curve (a numerical integral) was computed by combining spline interpolation and integration with the formula shown below:

(4) $$AUC=\int_{a}^{b}f\left(x\right)dx$$

The higher the AUC value, the more T cell epitopes against MEpiV were conserved. Normalization of AUC values with respect to the baseline score of MEpiV vaccine was performed as the number of sequences varied across the years. This enabled a direct comparison of the epitope content conservation across the years.

### 2.7. Phylogenetic Analysis

The T cell epitope conservation was mapped onto a phylogenetic tree to correlate the T cell epitope conservation with a genetic evolution of swine IAV. Phylogenetic trees inferred from the maximum likelihood (ML) were constructed based on the HA protein (H1 and H3 subtypes) of circulating swine IAV strains with RAxML.v8 using the GTR-GAMMA nucleotide substitution model. Both phylogeny trees were rooted with midpoint. MEpiV vaccine epitopes were evaluated against H1 and H3 tree tips using the ggtree package version 2.2.4 in R [29].

## 3. Results

### 3.1. Swine IAV Dataset from 2013 to 2017

**Figure 2:**
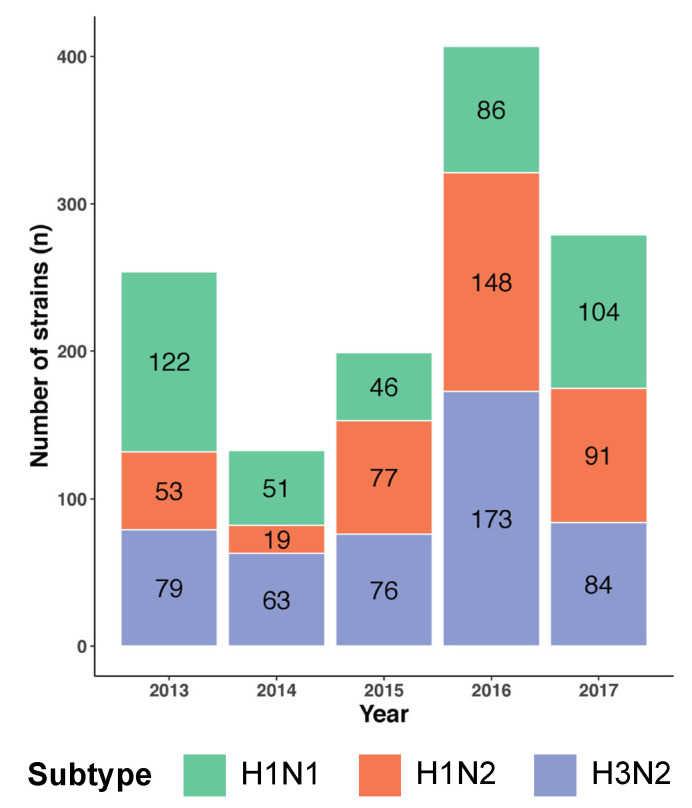
Swine IAV genome sequences from the H1N1, H1N2, and H3N2 subtypes from 2013–2017 included in this analysis. The color-coded stacked bar chart represents the three subtypes, each stacked component shows the number of strains per subtype for that year.

The goal of this study was to determine whether a vaccine designed in 2013 may continue to provide CMI boosting as was illustrated in 2019 [11]. The MEpiV vaccine contains 28 class I and 20 class II T cell epitopes and was produced as a plasmid DNA vaccine and tested in 2015 [8]. Circulating swine IAV whole genome sequences of three major subtypes (H1N1, H1N2, and H3N2) from 2013 to 2017 were computationally screened in the same stepwise process to evaluate their T cell epitope content in an epitope to epitope comparison to circulating strains (Figure 2). A total of 1272 whole genome swine influenza A sequences were analyzed, comprising 409 (32.2%) H1N1, 388 (30.5%) H1N2, and 475 (37.3%) H3N2 sequences. The highest number of sequences available was for 2016 (407 sequences; 32.0% of the total), while the lowest number was for 2014 (133 sequences; 10.5% of the total).

### 3.2. T Cell Epitope Content Comparison (EpiCC) of Swine MEpiV Vaccine against H1N1, H1N2, and H3N2 Circulating Swine IAV

In order to determine the conservation of MEpiV vaccine epitopes among the three circulating swine IAV subtypes, we applied EpiCC to facilitate a pairwise comparison of protein sequences. This sequence comparison method is based on an immunological property, potential T cell immunogenicity, rather than sequence identity. Shared (conserved) T cell epitopes between the vaccine target and the circulating swine IAV strains were assessed.

Higher EpiCC scores are thought to be associated with greater protection by vaccines against challenge strains [23]. For MEpiV vaccine class I epitopes, the highest EpiCC score is found for H1N1 swine IAVs (EpiCC score of 0.0256 with 98.5% conservation when normalized to the MEpiV baseline), and the lowest for H3N2 (EpiCC score of 0.0100 with 38.5% conservation when normalized to the MEpiV baseline). Interestingly, on average, EpiCC scores of MEpiV vaccine class II epitopes for all subtypes is 14.3% higher than scores of class I epitopes. The average range difference (in percentage) of class II EpiCC scores for all three subtypes is 25.6%, while for class I EpiCC scores it is 28.6%. The range difference of class II EpiCC scores is 11.7% smaller than the range difference of class I, indicating that the conservation of MEpiV class II epitopes was consistent in all of the circulating swine IAV subtypes that were analyzed. Detailed information for each of the circulating strains and their respective EpiCC scores are tabulated in Table S2.

**Figure 3:**
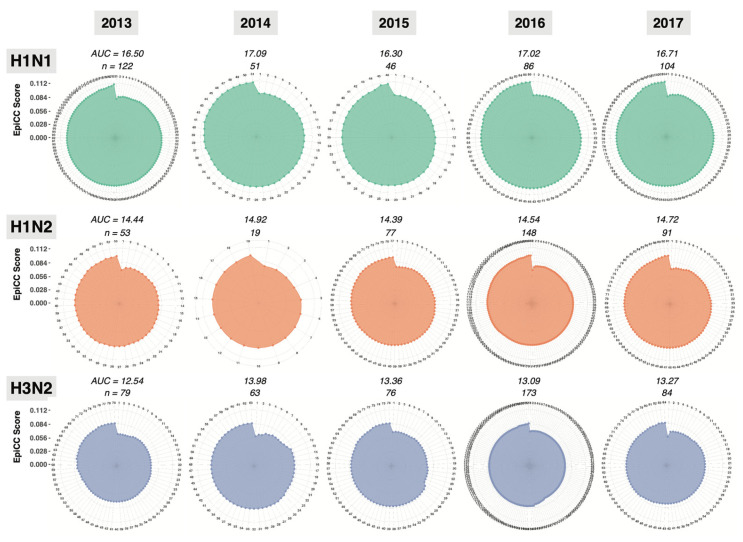
Radar plots enable the quantitative analysis of the degree of T cell epitope conservation between the conserved epitopes from all of the IAV proteins contained in a vaccine (here, MEpiV) and the epitopes from all of the IAV proteins contained in whole genome circulating strains for each year. The EpiCC score describing the T cell epitope conservation between the vaccine (MEpiV) against each swine IAV circulating strain is plotted on the radiating axes of radar plot for each year, for a period of 5 years, left to right. Circulating IAV strains were sorted from the lowest to the highest EpiCC scores. Radar plots for class II EpiCC scores are shown here and radar plots for class I are provided in supplemental data (Figure S1).

While detailed lists of EpiCC scores are informative, we also used radar plots to visualize the EpiCC scores. Radar plots were constructed to describe the degree of conservation of MEpiV vaccine class I and II T cell epitopes in the three prevalent swine flu subtypes (Figure 3) and the area under the curve for the EpiCC scores (AUC, outlined in color in Figure 3) was used to quantify and compare the T cell epitope conservation between the vaccine and circulating swine IAV each year. As shown in Figure 3 and Figure S1, the AUC described by the EpiCC scores is greater for the MEpiV vaccine against H1N1, than the AUC for H3N2 and H1N2 circulating strains. Thus, the vaccine is predicted to be effective against all circulating H1N1 strains in 2013 to 2017. The MEpiV vaccine is predicted to drive a broad CD4 immune response based on data published by Gutierrez et al. [24] and Hewitt et al. [25].

**Figure 4:**
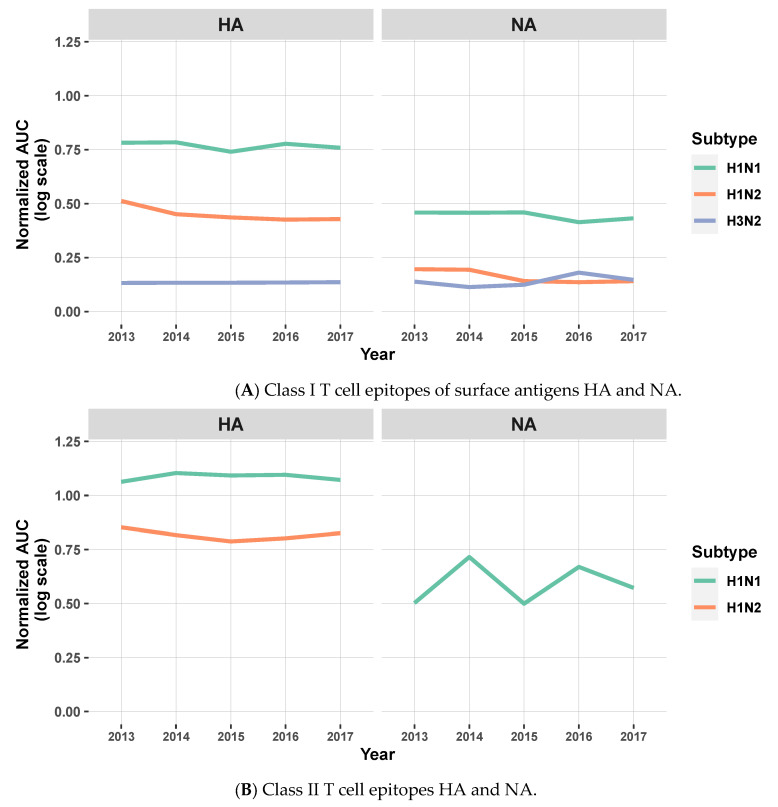
Line plots showing the normalized AUC for the comparison of MEpiV vaccine epitopes to epitopes found in circulating IAV strains for surface antigens HA and NA, by subtypes and by year for class I SLA (**A**) and class II SLA (**B**) epitopes. The AUC is shown on a normalized scale to enable the direct comparison by antigens, subtypes, years, and T cell epitopes classes. The lines that represent H1N2 (NA) and H3N2 (HA and NA) were removed in (**B**) as they showed no conservation. Similar line plots (different *y*-axis scaling) for the internal antigens are shown in Figure S2.

Computing the AUC facilitates the qualitative comparison of the vaccine against circulating strains over time. As expected, when considering the MEpiV computer-designed vaccine epitopes, the overall EpiCC scores, compared to circulating viral strains, did not change very much over time. The overall conservation was maintained for all three viral subtypes, although the total EpiCC scores were lower for H1N2 and H3N2 strains. Class II T cell epitopes were 80.8% more conserved on average, as compared to class I in all subtypes (Figure S1). We visualized these data on the individual antigen level in biaxial plots with the *x*-axis representing time and the *y*-axis representing the AUC for vaccine against circulating strains for that year (Figure 4). For the HA antigen, there was 79.5% conservation of MEpiV vaccine (both class I and II HA epitopes) in H1N1 over multiple years, whereas the HA epitope conservation in H1N2 and H3N2 were 51.7% and 8.6%, respectively.

The overall conservation of NA class I and II epitopes in H1N1 was 45.0%, while the conservation of vaccine epitopes in H1N2 and H3N2 strains was lower at 10.3% and 9.0%, respectively. The conservation in H1N2 and H3N2 for surface antigens was relatively low compared to H1N1, due to the complete lack of conservation (AUC of zero) for H3 and N2 epitopes in the MEpiV vaccine. Internal antigen epitopes were also well conserved across all subtypes (Figure S2), suggesting that internal proteins might contribute to vaccine efficacy. While the original MEpiV epitopes were selected from seven representative swine influenza strains, this finding suggests that vaccine epitopes that are highly conserved in one set of sequences for a given year may still be relevant and provide cross-protective immunity in the years that follow.

### 3.3. T Cell Epitope Conservation Analysis of Individual Epitopes Using Janusmatrix (JMX)

The EpiCC tool gives an overall score for the combined epitope content, rather than assessing and reporting on each epitope in a vaccine. Since the MEpiV is composed of distinct T cell epitopes, we wished to determine the conservation of each epitope over time, and therefore we performed an additional epitope-by-epitope analysis using JMX comparing the vaccine epitopes with their homolog in circulating strains. In this case, JMX searches the circulating swine IAV strains for 9-mers with the same TCR-facing amino acids as those of the input class I and II MEpiV vaccine epitopes [22]. The JMX homology score was calculated for every input MEpiV vaccine epitope that appears “homologous” to a given TCR, even though there may be minor variations in the MHC binding residues, as long as the peptide would still be predicted to bind to the same MHC.

While performing the JMX analysis to compare vaccine epitopes to circulating strain epitopes was matched for binding to the same MHC and identical at the TCR-face, we were able to identify the specific TCR-homologous 9-mers in circulating swine IAV strains. We applied JMX homology scores to further examine the level of conservation of individual T cell epitopes in every swine IAV subtype and quantify the overall conservation (Table 1).

Doing so, we were able to identify the most highly conserved T cell epitopes. Among 28 class I peptides, 16 of the peptides were more than 80% conserved in the three-circulating swine IAV subtypes throughout the 5-year period (Table 1A). Only two surface epitopes from NA were conserved and were N1-specific. Most of the highly conserved peptides were from internal antigens: PB2 (GTEKLTITY), PB1 (VSDGGPNLY, DTVNRTHQY), PA (QVSRPMFLY), NP (AFDERRNKY, CTELKLSDY, ASQGTKRSY, KSCINRCFY, DTVHDRTPY), and M1 (SLLTEVETY, LTEVETYVL, DLLENLQAY, LASCMGLIY, LASCMGLIY, NTDLEALME). The two most conserved peptides were SLLTEVETY and LTEVETYVL from the M1 protein. These peptides were found in all of the 1272 IAV strains. Interestingly, the least conserved peptides (GAKEVALSY and NMDKAVKLY) are also from M1, with conservation less than 3% in all of the subtypes and only being observed in 2013.

In addition, 10 out of 20 class II peptides were highly conserved (>80%) in circulating swine IAV strains (Table 1B). None of these highly conserved peptides were found in HA and NA, rather they were found in internal antigens such as PB1 (MMGMFNMLSTVLGVSI, YRYGFVANFSMELPSFGVSG), PA (EVHIYYLEKANKIKSEKTHIHIF, RSKFLLMDALKLSIEDP), NP (IEDLIFLARSALILRGSVAHKSCLP), M1 (TRQMVHAMRTIGTHPSSSA, TYVLSIIPSGPLKAEIAQRLESV, SCMGLIYNRMGTVTTEAAFGLVC), and NS2 (FEQITFMQALQLLLEVE, FQDILMRMSKMQLGSSSE). This suggests that epitopes from the internal antigens are well-conserved across strains and over time may contribute to vaccine efficacy.

**Figure 5:**
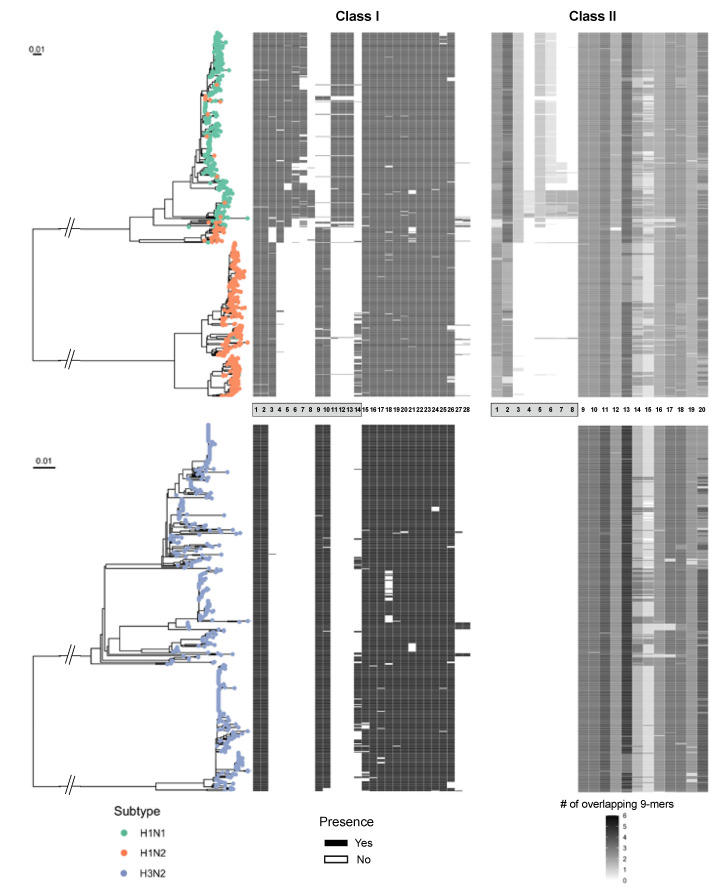
Phylogenetic tree of circulating swine H1 and H3 subtype IAV strains with predicted epitopes mapped to the tree tips. Class I and class II of MEpiV vaccine epitopes are shown in the heatmaps aligned with each associated strain. HA subtypes were color-coded. MEpiV epitopes were listed in the central panel sorted by an external (grey box) and internal proteins arrangement (numberings refer to Table 1). The black and white bars mapped adjacent to the phylogeny tree show the presence or absence of respective MEpiV vaccine epitopes in these circulating swine IAV strains.

Then, we used this epitope matching information generated from the JMX analysis jointly with HA phylogeny trees to visualize the distribution of MEpiV vaccine class I and II epitopes (Figure 5). Epitopes from both classes were well conserved in most internal proteins, as indicated by the presence of small bars adjacent to the tips of the respective HA phylogeny tree. Epitopes in the external proteins such as HA and NA are subtype-specific, demonstrating that the MEpiV vaccine consists of H1, N1, and N2-specific epitopes. A big blank under HA for the H3 phylogeny tree shows almost an absence of H3 epitopes in the MEpiV vaccine. Interestingly, although there are subtype-specific epitopes, we would expect that H1N1 and H1N2 IAV strains have a shared conservation in HA epitopes, however, H1-specific epitopes are only found conserved in 47 H1N2 swine IAV strains that are of the same clade as H1N1 IAV strains. Six out of eight class I and half of four class II HA epitopes were absent in the H1N2 swine IAV subtype.

### 3.4. Strains Identification for Conserved Peptides

The epitope to epitope-based comparison can also be used to identify strains that have the most or the least conserved T cell epitopes (Table S3), which may be important when selecting strains for a recombinant or inactivated whole antigen vaccine. Forty-four IAV sequences were shown to be highly conserved against the MEpiV prototype vaccine, with conservation at 75%. The majority of the sequences (42/44) belong to the H1N1 subtype, while two belong to the H1N2 subtype. In contrast, four swine IAV sequences had very few epitopes conserved with the prototype vaccine (46.4%); all of these strains were H3N2 subtypes. This is expected as most of the T cell epitopes included in MEpiV were HA H1-specific, and conservation across subtypes is not optimal, indicating that truly universal vaccines must include epitopes from more than one subtype.

This study demonstrates how EpiCC and JMX can be applied in complement for surveillance and analysis of epitope evolution and/or escape. One of the direct applications of the EpiCC program is to enable the selection of challenge IAV strains for vaccine studies. Furthermore, this work also serves a retrospective analysis that provides a baseline strain coverage estimate for MEpiV, but can easily be applied to other (new or old) vaccines against large numbers of new viruses.

### 3.5. Comparison of MEpiV and Commercial Swine Flu Vaccine

Immunity induced by inactivated virus vaccines usually wanes over time when it is no longer a close match to the circulating strains. To further investigate whether the T cell epitope conservation in a vaccine that was computationally designed to contain such epitopes was advantageous as compared to commercial swine flu vaccines, we compared the AUC computed from the EpiCC analysis for HA antigens of the MEpiV vaccine and the HA found in a commercial vaccine which comprises four HA vaccine strains of the major swine IAV seasonal subtypes, one H1N1, one H1N2, and two H3N2. The H1 components of these commercial vaccines were included since 2011, while the H3 components were introduced in 2016.

**Figure 6:**
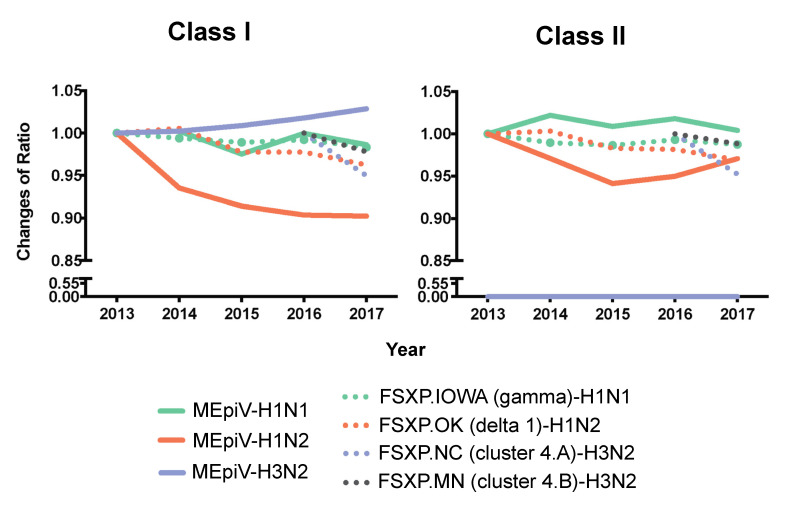
MEpiV compared to the commercial seasonal vaccine. The EpiCC analysis for the HA epitopes in MEpiV and HA from commercial (inactivated, whole) vaccines were calculated and then normalized to the EpiCC AUC determined for circulating strains for the vaccine in 2013, to show changes in AUC over time. The solid lines represent the HA antigen of MEpiV from an epitope-based vaccine and the dotted lines illustrate the HA components from a commercial swine vaccine.

A year-to-year comparison was made relative to 2013 for HA antigens of all the vaccine strains except for the H3 components of the commercial vaccine strains that were introduced in 2016. Changes in the conservation of the vaccines against the baseline year were calculated as a ratio, meaning that a score of 1.00 would indicate no change in the T cell epitope conservation (in AUC values), greater than 1.00 indicates an increasing T cell epitope conservation relative to 2013, and a ratio less than 1.00 implies a loss of T cell epitope conservation. The ratio of T cell epitope content (class I and II) for MEpiV over time, remains consistent or increases (except for H1N2) (Figure 6). Specifically, the H3 HA class I epitopes in the MEpiV vaccine showed a gradual increase of conservation in circulating swine IAV strains. In contrast, the ratio of conservation for the H3 conventional vaccine strains (FSXP.NC and FSXP.MN) decline over time. The same trend for FSXP.NC and FSXP.MN were observed in class II, however, there was no change in the class II epitope conservation for the MEpiV vaccine, as there were no H3-specific class II epitopes selected for the MEpiV vaccine. This result is consistent with the EpiCC and JMX analyses shown above.

## 4. Discussion

In general, vaccine efficacy assessment methods are lacking for swine IAV. More specifically, in lieu of challenge studies, there is no method available for evaluating new vaccines against circulating strains for cross-protection by T cell epitopes. Here, we used the EpiCC tool to approximate the potential T cell epitope cross-protection between MEpiV and circulating strains. In previously published studies, we established a threshold of cross-conservative epitope protection, using EpiCC to compare one vaccine against IAV strains circulating in 1 year [23]. We have also demonstrated the utility of EpiCC tool applied for another pathogen, Porcine circovirus 2 (PCV2) in a study evaluating multiple vaccines against circulating PCV2 strains [30]. In this study, we demonstrate how EpiCC can be used for the longitudinal analysis against evolving strains circulating in swine populations.

The current analysis applies the EpiCC tool to a computationally designed T cell epitope vaccine and compares the vaccine with circulating strains over a 5-year period. Having established the longitudinal conservation of the H1N1 T cell epitopes in the subunit vaccine, we then compared the 5-year trajectory of the epitope vaccine with that of a typical commercial swine IAV vaccine. The MEpiV retained conservation of T cell epitope content over time. This was especially true for seven T cell epitopes that were previously confirmed as immunogenic in a previous study [24]. In contrast with MEpiV, the antigenic ‘drift’ was evident for the commercial vaccine, resulting in lower EpiCC scores for the epitopes contained in the HA antigen over time, as expected. Consistency of the area under the curve (AUC) over years (for the MEpiV) suggests that the T cell epitopes in the prototype vaccine could reliably drive robust immune responses in swine regardless of the drift, and that a conserved epitope-driven vaccine may be a valuable adjunct to vaccination with whole, inactivated seasonal vaccine, as was shown by Hewitt et al. [25].

Comparing the T cell epitope conservation can contribute to assessing the projected efficacy of a vaccine. This study illustrates how EpiCC might be applied to evaluate several different vaccines, and to select the best vaccine strain (based on the T cell epitope conservation) for any given year. This is as relevant for IAV as it may be relevant for other emerging viruses such as COVID-19.

The analysis also demonstrates the use of JMX, a novel tool that searches for conserved T cell epitopes using TCR facing residues. JMX may make more accurate comparisons between T cell epitopes contained in vaccines as compared to circulating strains over time. By quantifying the conservation using JMX, we are also able to examine which T cell epitopes are conserved in which strains of IAV. This type of analysis may be useful for the selection of challenge strains in vaccine studies. Not surprisingly, epitopes from M1 and PB1 proteins were better conserved with circulating strain epitopes over the 5-year period studied in this example, and as expected, epitopes from HA and NA protein were much less conserved.

Compared to the commercial whole antigen killed vaccine, MEpiV T cell epitopes were highly conserved over time. This finding is particularly relevant for influenza, since cross-reactive antibodies may not be present when influenza strains shift, rather than drift [31]. Experts in the field have advocated for the development of ‘universal influenza vaccines’ that can boost immune responses in the absence of antibody cross-reactivity for this reason. The fact that lower levels of conservation were observed for H1N2 and H3N2 over time suggests that conserved epitope-based vaccines should be designed for each IAV subtype. We have explored the use of MEpiV-type vaccines given by the heterologous prime-boost with a commercial swine influenza vaccine (which contains a whole HA antigen) and found increased immunogenicity by priming with the MEpiV vaccine over the homologous commercial vaccine prime-boost, an equivalent body temperature control 1 day after the pH1N1 challenge, and reduced lung lesions and influenza antigen, as illustrated by Hewitt et al. [25]. Reducing the overall viral burden and increasing the average daily gain, distributed across large populations of swine, may prove cost-effective for pork producers. One application of ‘universal’ T cell epitope-based vaccines being explored in humans is to combine them with seasonal vaccines, a topic which might also be of interest to the animal health community [32,33,34].

Moreover, we note that the SLA alleles selected for this study were reported as prevalent in outbred swine populations [27,28] and on low-resolution haplotyping results in a small number of pigs [24]. We considered this set of alleles a first proxy for commonly expressed alleles. However, these alleles might not represent the complete SLA diversity or the most prevalent alleles in the US swine outbreed population. While EpiCC scores might be different, T cell epitope predictions for highly prevalent haplotypes that represent a large percentage of the US swine population will likely produce more relevant results. Systematic studies to investigate the distribution of SLA haplotypes in outbred populations of pigs in the US will have a significant impact on our ability to develop prediction models for a more comprehensive set of SLA alleles.

## 5. Conclusions

In conclusion, we applied the EpiCC tool to enable research on the impact of conserved T cell epitope-based vaccines as compared to whole antigen vaccines for influenza and other pathogens. As shown here, the EpiCC tool enables a comparison between vaccines and circulating field strains, but it is also useful for identifying whether existing vaccines might have efficacy (at the T cell epitope level) against an emerging infection. The EpiCC tool is likely to be useful for application to other viral populations such as emerging G4 influenza, African Swine Fever, as well as to human influenza strains versus candidate vaccines.

## 6. Patents

The MEPiV vaccine design is an intellectual property that is shared between EpiVax and the University of Rhode Island.

## Figures and Tables

**Table:** (**A**)

No.	Antigen	Class I Epitopes	JMX Homology Score (% of Conservation)			Average Conservation (%)
			H1N1	H1N2	H3N2	
1	HA	GMVDGWYGY	401.7 (98.2)	384.8 (99.2)	240.7 (50.7)	79.0
2	HA	GMIDGWYGY	401.7 (98.2)	384.8 (99.2)	240.7 (50.7)	79.0
3	HA	SVKNGTYDY	402.8 (98.5)	308.0 (79.4)	0.5 (0.1)	9.2
4	HA	RIYQILAIY	392.8 (96.0)	57.0 (14.7)	0	37.6
5	HA	NADTLCIGY	375.0 (91.7)	22.0 (5.7)	0	22.9
6	HA	TSADQQSLY	352.0 (86.1)	17.0 (4.4)	0	19.5
7	HA	LSTASSWSY	306.5 (74.9)	16.5 (4.3)	0	17.9
8	HA	ITIGKCPKY	58.8 (14.4)	3.5 (0.9)	0	3.6
9	NA	KSCINRCFY	0	384.0 (99.0)	474.0 (99.8)	99.4
10	NA	DTVHDRTPY	0	371.3 (95.7)	468.0 (98.2)	96.9
11	NA	GTIKDRSPY	322.25 (78.8)	0	0	78.8
12	NA	EMNAPNYHY	337.29 (82.5)	0	0	82.5
13	NA	ELDAPNYHY	381.86 (93.4)	0	0	93.4
14	NA	EICPKLAEY	0	98.4 (25.4)	120.6 (25.4)	25.4
15	PB2	GTEKLTITY	405.7 (99.2)	379.7 (97.9)	454.7 (95.7)	97.6
16	PB1	VSDGGPNLY	408.4 (99.9)	386.2 (99.5)	472.0 (99.4)	99.6
17	PB1	DTVNRTHQY	409.0 (100.0)	386.7 (99.7)	468.0 (98.5)	99.4
18	PA	QVSRPMFLY	400.6 (97.9)	379.6 (97.8)	433.0 (91.2)	95.7
19	NP	AFDERRNKY	407.8 (99.7)	386.5 (99.6)	471.8 (99.3)	99.5
20	NP	CTELKLSDY	406.0 (99.3)	383.5 (98.8)	472.0 (99.4)	99.2
21	NP	ASQGTKRSY	400.0 (97.8)	369.0 (95.1)	464.0 (97.7)	96.9
22	M1	SLLTEVETY	409 (100.0)	388 (100.0)	475.0 (100.0)	100.0
23	M1	LTEVETYVL	409 (100.0)	388 (100.0)	475.0 (100.0)	100.0
24	M1	DLLENLQAY	407 (99.5)	387 (99.7)	468.0 (98.5)	99.2
25	M1	LASCMGLIY	399 (97.6)	388 (100.0)	473.0 (99.6)	99.1
26	M1	NTDLEALME	399 (97.6)	366 (94.3)	463.0 (97.5)	96.5
27	M1	NMDKAVKLY	11 (2.7)	10 (2.6)	16.0 (3.4)	2.9
28	M1	GAKEVALSY	12 (2.9)	9 (2.3)	13.0 (2.7)	2.6

**Table:** (**B**)

No.	Antigen	Class II Epitopes	JMX Homology Score (% of Conservation)			Average Conservation (%)
			H1N1	H1N2	H3N2	
1	HA	YEELREQLSSVSSFER	392.6 (96.0)	365.3 (94.1)	0	63.4
2	HA	STRIYQILAIYSTVASSLVLV	393.1 (96.1)	253.4 (65.3)	0	53.8
3	HA	GDKITFEATGNLVVPRY	348.2 (85.1)	56.4 (14.5)	0	33.2
4	HA	VPRYAFAMERNAGSGIIIS	13.0 (3.2)	1.1 (0.3)	0	1.2
5	NA	CRTFFLTQGALLNDKH	408.4 (99.9)	0	0	33.3
6	NA	SVVSVKLAGNSSLCPV	102.9 (25.2)	0	0	8.4
7	NA	NQTYVNISNTNFAAGQSVVSVKL	66.0 (16.1)	0	0	5.4
8	NA	MANLILQIGNIISIWISHS	62.1 (15.2)	0	0	5.1
9	PB1	MMGMFNMLSTVLGVSI	409.0 (100.0)	387.4 (99.8)	474.9 (100.0)	99.9
10	PB1	YRYGFVANFSMELPSFGVSG	409.0 (100.0)	388.0 (100.0)	474.5 (100.0)	100.0
11	PA	EVHIYYLEKANKIKSEKTHIHIF	406.3 (99.3)	386.1 (99.5)	472.9 (99.6)	99.5
12	PA	RSKFLLMDALKLSIEDP	405.9 (99.2)	381.0 (98.2)	474.7 (99.9)	99.1
13	NP	IEDLIFLARSALILRGSVAHKSCLP	400.3 (97.9)	311.3 (80.2)	454.3 (95.6)	91.2
14	NP	TRGVQIASNENVETMDSNTLELR	346.5 (84.7)	243.8 (62.8)	268.5 (56.5)	68.0
15	NP	IDPFKLLQNSQVVSLMRP	343.4 (84.0)	270.6 (69.7)	296.9 (62.5)	72.1
16	M1	TRQMVHAMRTIGTHPSSSA	398.6 (97.5)	380.8 (98.1)	462.1 (97.3)	97.6
17	M1	SCMGLIYNRMGTVTTEAAFGLVC	399.3 (97.6)	382.0 (98.5)	462.7 (97.4)	97.8
18	M1	TYVLSIIPSGPLKAEIAQRLESV	395.3 (96.7)	367.2 (94.7)	465.5 (98.0)	96.5
19	NS2	FEQITFMQALQLLLEVE	407.6 (99.7)	384.9 (99.2)	466.6 (98.2)	99.0
20	NS2	FQDILMRMSKMQLGSSSE	364.9 (89.2)	327.2 (84.3)	358.9 (75.6)	83.0

## Data Availability

Not applicable.

## Supplementary Materials

The following are available online at https://www.mdpi.com/article/10.3390/vaccines9050468/s1. Figure S1: Radar plots showing the degree of class I T cell epitope conservation between the conserved epitopes in the MEpiV for the HA antigen and epitopes contained in HA from circulating strains for each year; Figure S2: Line plots showing the degree of conservation by antigens, subtypes, and years; Table S1: MEpiV vaccine class I and II peptides; Table S2: EpiCC scores of all swine IAV circulating strains; Table S3: List of swine IAV circulating strains that have the most and the least conserved class I (A) and II (B) epitopes.
