# Supplementary material for: Quantifying the Persistence of Vaccine-Related T Cell Epitopes in Circulating Swine Influenza A Strains from 2013–2017
Source: Vaccines (Basel). 2021 May 6;9(5):468. doi: 10.3390/vaccines9050468 (PMC8148565; doi:10.3390/vaccines9050468)
Supplement: Supplementary file 1 [file vaccines-09-00468-s001.zip › vaccines-1161983-sup.pdf]

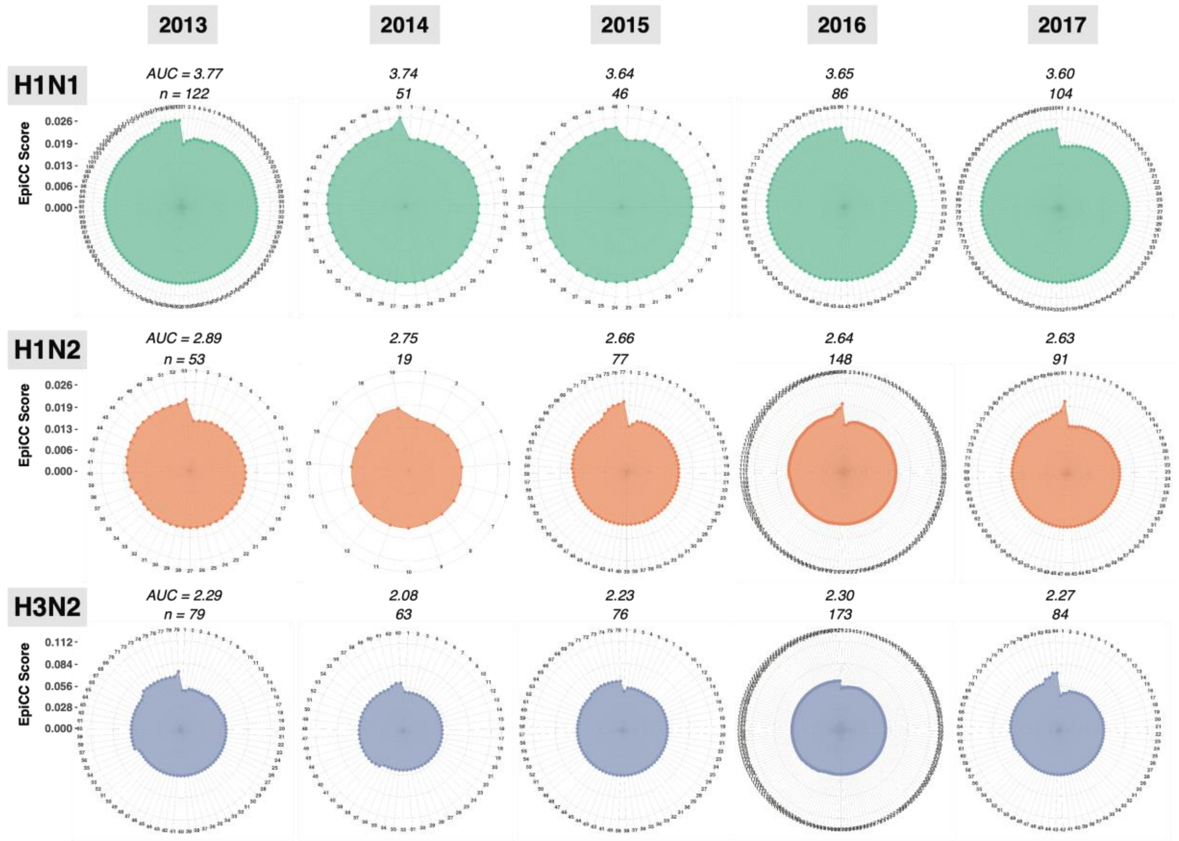

Figure S1. Radar plots showing degree of class I T cell epitope conservation between the conserved epitopes in the MEpiV for the HA antigen and epitopes contained in HA from circulating strains for each year. The radar plots show T cell epitope conservation between the T cell-directed multi-epitope DNA vaccine against each swine IAV circulating strain (axes of radar plot) over five years. The strains were sorted from lowest to highest EpiCC scores. The multi-epitope DNA vaccine is predicted to drive better CD4 immune response based on data published in Gutierrez 2017 and Hewitt 2019.

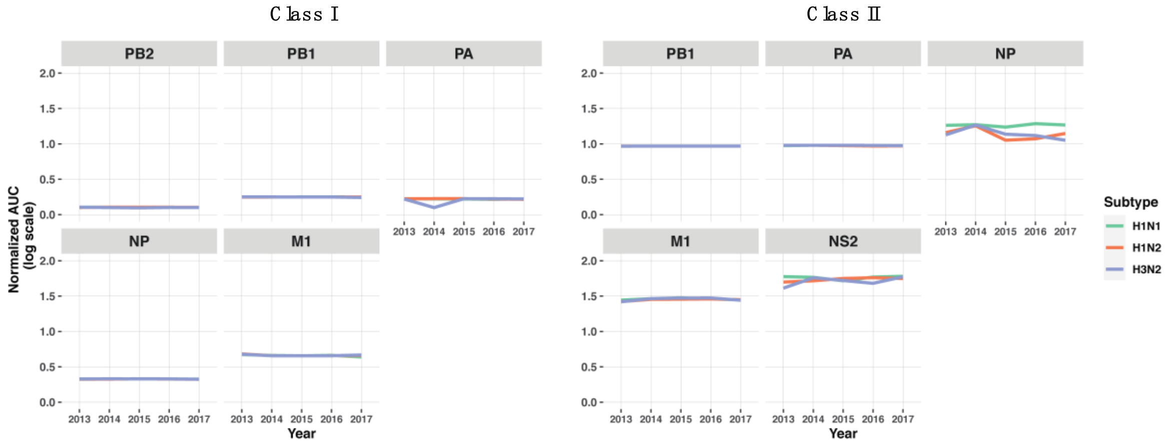

Figure S2. Line plots showing the degree of conservation by antigens, subtypes and years. The degree of conservation (AUC) is normalized to ease direct comparison by internal antigens, subtypes, years and T cell epitopes classes.

Table S1. MEpiV vaccine class I and II peptides.

Antigen	Class I peptide	Class II peptide
PB2	GTEKLTITY	-
PB1	VSDGGPNLY<br>DTVNRTHQY	MMGMFNMLSTVLGVSI<br>YRYGFVANFSMELPSFGVSG
PA	QVSRPMFLY	EVHIYYLEKANKIKSEKTHIHIF<br>RSKFLLMDALKLSIEDP
HA	GMVDGWYGY<br>GMIDGWYGY<br>SVKNGTYDY<br>RIYQILAIY<br>NADTLCIGY<br>TSADQQSLY<br>LSTASSWSY<br>ITIGKCPKY	YEELREQLSSVSSFER<br>STRIYQILAIYSTVASSLVLV<br>GDKITFEATGNLVVPRY<br>VPRYAFAMERNAGSGIIIS
NP	AFDERRNKY<br>CTELKLSDY<br>ASQGTKRSY	IEDLIFLARSALILRGSVAHKSCLP<br>TRGVQIASNENVETMDSNTLELR<br>IDPFKLLQNSQVVSLMRP
NA	KSCINRCFY<br>DTVHDRTPY<br>GTIKDRSPY<br>EMNAPNYHY<br>ELDAPNYHY<br>EICPKLAEY	CRTFFLTQGALLNDKH<br>SVVSVKLAGNSSLCPV<br>NQTYVNISNTNFAAGQSVVSVKL<br>MANLILQIGNIISIWISHS
M1	SLLTEVETY<br>LTEVETYVL<br>DLLENLQAY<br>LASCMGLIY<br>NTDLEALME<br>NMDKAVKLY<br>GAKEVALSY	TRQMVHAMRTIGTHPSSSA<br>SCMGLIYNRMGTVTTEAAFGLVC<br>TYVLSIIPSGPLKAEIAQRLESV
NS2	-	FEQITFMQALQLLLEVE<br>FQDILMRMSKMQLGSSSE

Table S2. EpiCC scores of all swine IAV circulating strains (in Excel).

Table S3. List of swine IAV circulating strains that have the most and the least conserved class I (**A**) and II (**B**) epitopes.

**(A) Class I most conserved strains against the vaccine. Conservation at 75% (21/28)**		
**No.**	**Strain Name**	**Subtype**
1	A/Swine/Arkansas/D0386/2013	H1N1
2	A/Swine/Minnesota/A01392911/2013	
3	A/Swine/Minnesota/A01394863/2013	
4	A/Swine/Minnesota/MT1301S79/2013	
5	A/Swine/Ohio/A01349978/2013	
6	A/Swine/Ohio/A01432602/2013	
7	A/Swine/Illinois/A01490609/2014	
8	A/Swine/Illinois/A01492501/2014	
9	A/Swine/Illinois/A01493472/2014	
10	A/Swine/Iowa/A01410472/2014	
11	A/Swine/Kansas/A01410327/2014	
12	A/Swine/Minnesota/A01491447/2014	
13	A/Swine/Minnesota/A01491704/2014	
14	A/Swine/Missouri/A01492887/2014	
15	A/Swine/Nebraska/A01366774/2014	
16	A/Swine/Nebraska/A01491300/2014	
17	A/Swine/Nebraska/A01492657/2014	
18	A/Swine/Nebraska/A01566172/2014	
19	A/Swine/North Carolina/A01410573/2014	
20	A/Swine/Oklahoma/A01410195/2014	
21	A/Swine/Oklahoma/A01476227/2014	
22	A/Swine/Indiana/A01260972/2015	
23	A/Swine/Illinois/A01729364/2016	
24	A/Swine/Illinois/A01749912/2016	
25	A/Swine/Illinois/A01749913/2016	
26	A/Swine/Illinois/A01749914/2016	

27	A/Swine/Illinois/A01775937/2016	
28	A/Swine/Illinois/A01776206/2016	
29	A/Swine/Illinois/A01777039/2016	
30	A/Swine/Illinois/A01778882/2016	
31	A/Swine/Indiana/16TOSU4933/2016	
32	A/Swine/Indiana/A01671620/2016	
33	A/Swine/Indiana/A01812242/2016	
34	A/Swine/Iowa/A01781047/2016	
35	A/Swine/Iowa/A01782230/2016	
36	A/Swine/Missouri/A01775100/2016	
37	A/Swine/Missouri/A01775109/2016	
38	A/Swine/Nebraska/A01783006/2016	
39	A/Swine/Ohio/A01104092/2016	
40	A/Swine/Tennessee/A01894329/2016	
41	A/Swine/Iowa/A02214835/2017	
42	A/Swine/Kansas/A01378027/2017	
1	A/Swine/Indiana/16TOSU0646/2016	H1N2
2	A/Swine/Michigan/A01259077/2017	
**Class I least conserved strains against the vaccine. Conservation at 46.4% (13/28)**		
1	A/Swine/Kansas/A01377649/2015	H3N2
2	A/Swine/Missouri/A01840324/2015	
3	A/Swine/Florida/UF1/2017 A/Swine/Mis-	
4	souri/A02136832/2017	

**(B) Class II most conserved strains against the vaccine. Conservation at 32.3% (74/229)**		
**No.**	**Strain Name**	**Subtype**
1	A/Swine/Arkansas/D0386/2013	H1N1
2	A/Swine/Minnesota/A01381276/2013	
3	A/Swine/Ohio/A01349978/2013	
4	A/Swine/Ohio/A01432602/2013	
**Class II least conserved strains against the vaccine. Conservation at 15.3% (35/229)**		
1	A/Swine/Nebraska/A01493915/2014	H3N2
2	A/Swine/Florida/UF1/2017	
